# Circulating Endocannabinoids in Canine Multicentric Lymphoma Patients

**DOI:** 10.3389/fvets.2022.828095

**Published:** 2022-02-15

**Authors:** Jennifer K. Hay, Samuel E. Hocker, Gabrielle Monteith, J. Paul Woods

**Affiliations:** ^1^Department of Clinical Sciences, College of Veterinary Medicine, Kansas State University, Manhattan, KS, United States; ^2^Department of Clinical Studies, Ontario Veterinary College, University of Guelph, Guelph, ON, Canada

**Keywords:** biomarker, dog, 2-arachidonoylglycerol, N-arachidonoylethanolamine, oleoythanolamide, palmitoylethanolamide

## Abstract

The endocannabinoid system is increasingly being implicated in the pathogenesis and progression of various human cancers. Specifically, increased levels of 2-arachidonoylglycerol (2-AG) and oleoythanolamide (OEA) have been demonstrated in human diffuse large B-cell lymphoma (DLBCL) and chronic lymphocytic leukemia (CLL) patients, respectively. The objectives of this paper were to compare 2-AG, OEA, N-arachidonoylethanolamine (AEA), and palmitoylethanolamide (PEA) levels between dogs with multicentric lymphoma and healthy control dogs. In addition, evaluate 2-AG, OEA, AEA, and PEA levels as biomarkers for progression free interval (PFI) and overall survival time (OST) in the dogs with lymphoma. The study consisted of 26 dogs with multicentric B cell lymphoma, 14 dogs with multicentric T cell lymphoma, and 12 healthy control dogs. Serum 2-AG, OEA, AEA, and PEA levels were measured using liquid chromatography combined with tandem mass spectrometry (LC—MS/MS) in dogs with lymphoma and in healthy dogs. OEA, AEA, and PEA levels were significantly elevated in dogs with lymphoma compared to healthy controls (*p* < 0.05). Total AG was significantly higher in healthy control dogs (*p* = 0.049). There was no significant difference between dogs with B cell and T cell lymphoma for any of the measured endocannabinoids. Elevated PEA was significantly associated with decreased PFI (*p* = 0.04) in dogs with lymphoma with a hazards ratio of 1.816 [95% Confidence Interval (CI): 1.020–3.232]. Overall, dogs with lymphoma have elevated levels of OEA, AEA, and PEA. PEA levels have the potential to be a prognostic biomarker.

## Introduction

Canine lymphoma (LSA) is the most common hematopoietic neoplasm in dogs and shares many biological and therapeutic similarities to Non-Hodgkin lymphoma in humans (1–5). Numerous prognostic factors and biomarkers such as immunophenotype, WHO clinical stage, anatomic location, serum thymidine kinase 1, and lactate dehydrogenase have been evaluated in canine LSA. Much information has been elucidated about canine LSA; however, further understanding of factors associated with disease progression and prognosis is needed (6–13).

The reprogramming of metabolic pathways in tumor cells has gained increased attention in recent years and there is increasing evidence for lipid metabolism dysregulation in various cancers (14–16). The endocannabinoid system (ECS) consists of endogenous lipid endocannabinoids N-arachidonoethylethanolamine (AEA) and 2-arachidonoylglycerol (2-AG), and their G-protein coupled cannabinoid receptors cannabinoid receptor 1 (CB1) and cannabinoid receptor 2 (CB2). Additionally, there are structurally related endogenous fatty acid compounds including palmitoylethanolamide (PEA) and oleoythanolamide (OEA) that facilitate the actions of endocannabinoids and directly activate other receptors, including the peroxisome proliferator-activated receptor-α (PPAR-α) (17, 18). There are also enzymes that synthesize [diacylglycerol (DAGL), N-acylphosphatidyl-ethanolamine phospholipase D (NAPE-PLD)] and degrade [fatty acid amine hydrolase (FAAH), monoaceylglycerol lipase (MAGL)] endogenous cannabinoids to regulate their levels *in vivo* (19). The endocannabinoid system has many well-known regulatory functions in the body including neuromodulatory activity, energy metabolism, and immunity (19).

There has been increasing interest in the endocannabinoid system with respect to its application in oncology. Alterations of the endocannabinoids and their receptors have been documented in a variety of cancers and these alterations have been linked to disease progression and outcome (19, 20). A prospective study comparing serum endocannabinoids between humans with diffuse large B cell lymphoma (DLBCL) and healthy controls found elevated levels of 2-AG in the DLBCL patients. In addition, treatment of DLBCL cell lines with exogenous 2-AG had a proliferative effect on a subset suggesting that 2-AG may have a role in the pathogenesis or progression of certain DLBCLs (14). A study evaluating endocannabinoids in people with chronic lymphocytic leukemia (CLL) found high levels of OEA in the plasma of CLL patients compared with normal donors and showed that OEA significantly increased the viability of CLL cells *in vitro* (15).

To date, there have been no studies investigating circulating endocannabinoids in canine lymphoma patients or their effect on disease progression. Alterations in circulating endocannabinoids may represent a non-invasive prognostic biomarker and potentially a therapeutic target. The objective of this study was to compare circulating levels of 2-AG, OEA, AEA and PEA between dogs with multicentric lymphoma and normal healthy dogs. Additional objectives included comparing circulating levels of 2-AG, AEA, PEA, and OEA between dogs diagnosed with multicentric B-cell lymphoma vs. multicentric T-cell lymphoma; and to evaluate the prognostic value of circulating 2-AG, AEA, PEA, and OEA in canine multicentric lymphoma patients treated with a cyclophosphamide, doxorubicin, vincristine and prednisone (CHOP) chemotherapy protocol.

## Materials and Methods

### Study Design and Dog Selection

Serum from two groups of dogs was obtained for analysis. One group consisted of client-owned dogs with multicentric lymphoma that were presented to Mona Campbell Center for Animal Cancer, University of Guelph, Canada over a 4-year period (2015–2019). The serum samples were collected at the time of diagnosis and obtained from the University of Guelph's Institute for Comparative Cancer Investigation's Companion Animal Tumor Sample Bank. The serum was stored at −80°C until analysis. Dogs were eligible for inclusion if they had a cytologic or histologic diagnosis of multicentric lymphoma, immunophenotyping, and were treated with a 25 week CHOP-based chemotherapy protocol as their first line treatment. Exclusion criteria included dogs with indolent lymphoma, concurrent serious illness at the time of diagnosis, or inadequate follow up to determine their response to treatment. Initial staging tests included physical exam, complete blood count, serum biochemical profile, urinalysis, thoracic radiographs, and abdominal ultrasound. Tumor stage was designated based on the World Health Organization (WHO) criteria. Substage was assigned retrospectively based on the medical record with substage “a” defined as no clinical signs and substage “b” defined as clinical signs of illness including general malaise, respiratory disease or gastrointestinal disease. Breed, sex, weight, age, stage, substage, and immunophenotype were recorded for each dog. Response to therapy was based on the Veterinary Cooperative Oncology Group (VCOG) criteria for peripheral nodal lymphoma (1) as determined by measurement of peripheral lymph nodes with calipers. The other group was a control group of normal healthy dogs from the University of Guelph community. Dogs were considered healthy if they had no history or clinical signs of disease, normal minimum database (complete blood count, serum biochemistry, urinalysis), and were not currently prescribed medications at the time of sample collection. The samples were collected and were spun immediately after collection. They were spun at 2,000 × g for 10 min at 4°C and then stored at −80°C until analysis. The serum was stored at −80°C until analysis. This study was approved by the University of Guelph Animal Care Committee and written owner consent was obtained.

### Endocannabinoid Analysis

Fatty acid ethanolamines in the serum samples were quantified by LC-MS/MS at the Analytical Facility for Bioactive Molecules (The Hospital for Sick Children, Toronto, Canada). Serum samples (200 μL), standards (2-AG, OEA, AEA, PEA), and deuterated internal standards (AEA-d4, OEA-d4, PEA-d4, 2-AG-d5) were added to Eppendorf tubes and brought to a total volume of 1 mL with water. Tubes were vortexed and then loaded onto preconditioned (1 mL methanol then 1 mL water) Oasis HLB SPE tubes. The SPE tubes were washed twice with 1 mL of 40/60 methanol/water and then the fatty acid ethanolamines were eluted into conical tubes using 2 mL of acetonitrile. The acetonitrile was evaporated under a gentle flow of nitrogen gas and the residue reconstituted in 150 μL of acetonitrile. Samples were injected onto a Kinetex XB-C18 50 × 3.0 mm column on an Agilent 1290 LC system coupled to a Sciex Q-Trap 5500 mass spectrometer. Samples were eluted using a gradient of (A) 0.1% formic acid and (B) 0.1% formic acid in acetonitrile. Data was collected and analyzed using SCIEX Analyst v1.7. 2-AG was reported as total AG (consisting of 1-AG and 2-AG) due to isomerization during extraction (21).

Quantification of 2-AG is difficult to perform due to its instability in aqueous solutions, leading to rapid molecular rearrangement and formation of 1-arachidonoylglycerol (1-AG) which is its less biologically active form. Most methods assume that 2-AG and 1-AG both originate from biological 2-AG; therefore, total AG is the sum of 2-AG and 1-AG peaks acquired through chromatography (19, 22, 23).

### Evaluation of Treatment Response

Objective response rate (ORR) was the sum of patients with a complete or partial response after initiation of treatment divided by the total number of patients (24). Progression free interval (PFI) was defined as the time from initiation of the CHOP protocol until progressive disease. Overall survival time (OST) was defined as the time from initiation of the CHOP protocol to death from any cause. Dogs that were lost to follow up or still alive at the time of data analysis were censored.

### Statistical Analysis

Statistical analysis was performed using SAS software (SAS Institute Inc. 2013. SAS/STAT 9.4. Cary, NC: SAS Institute Inc.) A general linear mixed model was fit to determine significant differences in 2-AG, OEA, AEA, and PEA between groups (normal, pooled lymphoma, T cell, and B cell pooled), sex, age, and body weight as well as interactions were included in the model. Categorical data was expressed as percentages whereas continuous data was expressed as median and range. Models were then simplified removing effects at *p* > 0.15 to achieve a final model. Comparison of means were made at the average age for the groups when there was significant interaction of age with type. Data was checked for normality with a Shapiro-Wilk test and examination of the residuals. Data was log transformed to meet the assumptions of normality. *Post-hoc* group comparisons included a Tukey adjustment. Differences between groups were considered significant when the corresponding *p*-value was <0.05. A cox proportional hazard model accounting for censoring that included type, stage, age, and weight was modeled to determine if endocannabinoid levels influence progression free interval or overall survival time.

## Results

### Study Population

A total of 52 serum samples were analyzed including the serum from 26 dogs with multicentric B cell lymphoma, 14 dogs with multicentric T cell lymphoma, and 12 normal healthy dogs. Among the lymphoma patients there were a total of 19 breeds represented. Breeds with >2 dogs included mixed breed (*n* = 11), golden retriever (*n* = 4), and boxer (*n* = 4). Among the normal dogs there were a total of 8 breeds represented with mixed breed (*n* = 5) being the most common. Full staging was not performed in all dogs with lymphoma, thoracic radiographs were performed in 21 dogs (53%) and 17 dogs (43%) had an abdominal ultrasound. No dog had a bone marrow aspirate performed. Patient characteristics for the dogs with lymphoma and healthy control dogs are summarized in Table 1.

**Table 1:** Clinical characteristics of dogs with lymphoma and healthy controls.

	**Dogs with lymphoma—all** **(***n*** = 40)**	**Dogs with B cell lymphoma** **(***n*** = 26)**	**Dogs with T cell lymphoma** **(***n*** = 14)**	**Healthy control dogs** **(***n*** = 12)**
Age (years) median (range)	10.72 (5.57–16.85)	10.72 (5.57–16.85)	10.76 (6.85–14.71)	7.0 (3.95–11.87)
Body weight (kg) median (range)	28 (6.3–54)	28.8 (6.3–54)	25.6 (7.74–42.8)	14.66 (2.64–22.8)
**Sex**				
Female (spayed)	13 (13)	8 (8)	5 (5)	8 (8)
Male (castrated)	27 (24)	18 (16)	9 (8)	4 (4)
**WHO stage (%)**				
I	1 (3%)	0	1 (7%)	
II	4 (10%)	1 (4%)	3 (21%)	
III	24 (60%)	17 (65%)	7 (50%)	
IV	8 (20%)	6 (23%)	2 (14%)	
V	3 (8%)	2 (8%)	1 (7%)	
**Substage (%)**				
a	24 (60%)	19 (73%)	5 (36%)	
b	16 (40%)	7 (27%)	9 (64%)	

*WHO, World Health Organization*.

### Treatment Response

The overall objective response rate to the CHOP chemotherapy was 90% with an ORR of 100% for dogs with B cell lymphoma and 71% for dogs with T cell lymphoma. The outcome characteristics for the lymphoma populations are summarized in Table 2.

**Table 2:** Outcome characteristics for dogs with multicentric lymphoma.

	**Dogs with lymphoma (all)** **(***n*** = 40)**	**Dogs with B cell lymphoma** **(***n*** = 26)**	**Dogs with T cell lymphoma** **(***n*** = 14)**
**Response rate**			
CR	30 (75%)	22 (85%)	8 (57%)
PR	6 (15%)	4 (15%)	2 (14%)
SD	0	0	0
PD	4 (10%)	0	4 (29%)
ORR	90%	100%	71%
Completed CHOP	19 (48%)	16 (62%)	3 (21%)
PFI (days) median (range)	159 (14–524)	237 (31–524)	79.5 (14–385)
OST (days) median (range)	252 (24–1,236)	295 (50–1,236)	126 (24–364)

*CR, complete response; PR, partial response; SD, stable disease; PD, progressive disease; ORR, objective response rate; PFI, progression free interval; OST, overall survival time*.

### Serum Endocannabinoid Levels

Serum endocannabinoid levels were evaluated for significant associations with age, sex or body weight. Age was the only variable with significance for OEA, AEA, and PEA levels. As age increased, the endocannabinoid levels decreased in normal dogs and dogs with T cell lymphoma, whereas the levels increased as age increased in dogs with B cell lymphoma.

The median values of the endocannabinoids are in Table 3. AEA (*p* = 0.002), PEA (*p* = 0.026), and OEA (*p* < 0.0001) levels were all significantly higher in the serum of dogs with lymphoma compared to healthy control dogs. Additionally, AEA (*p* = 0.003), OEA (*p* = 0.0001), and PEA (*p* = 0.042) levels were significantly higher in dogs with B cell lymphoma compared to the healthy population, and AEA (*p* = 0.004) and OEA (*p* < 0.0001) were also significantly higher in dogs with T cell lymphoma compared to healthy controls. There was no significant difference between any of the measured serum endocannabinoids for dogs with B cell lymphoma when compared to dogs with T cell lymphoma.

**Table 3:** Serum AEA, OEA, PEA, and total AG measured in the control and lymphoma groups.

		**AEA (ng/mL)**	**OEA (ng/mL)**	**PEA (ng/mL)**	**Total AG (ng/mL)**
**Groups**
Healthy control dogs (*n* = 12)	Median	0.52	1.5	1.81	87.5
	Range	0.47–0.79	0.3–9.75	0.44–7.55	7.1–285
Dogs with lymphoma (all) (*n* = 40)	Median	0.64	3.63	1.81	24.2
	Range	0.53–1.32	1.76–10.45	0.91–3.31	7.25–600
Dogs with B cell lymphoma (*n* = 26)	Median	0.65	3.58	1.82	26.3
	Range	0.53–1.27	2.3–10.45	0.91–3.31	7.25–600
Dogs with T cell lymphoma (*n* = 14)	Median	0.63	4.70	1.69	18.03
	Range	0.54–1.32	1.76–5.4	0.95–2.12	7.25–182

*AEA, anandamide; OEA, oleoythanolamide; PEA, palmitoylethanolamide; total AG, total arachidonoylglycerol*.

Total AG was significantly higher in normal dogs when compared to dogs with lymphoma (*p* = 0.049) and was significantly higher in normal dogs compared to dogs with T cell lymphoma (*p* = 0.049). There was no significant difference in total AG between dogs with B cell and T cell lymphoma.

### Prognostic Evaluation

The median PFI for all lymphoma patients was 159 days, with a PFI of 237 days for dogs with B cell and 79.5 days for dogs with T cell immunophenotypes. Four dogs clinically maintained their initial remission and were censored from analysis. Of those, three were euthanized at 276, 339, and 919 days for congestive heart failure (*n* = 1) and neurologic disease (*n* = 2) respectively. One dog was still alive at the time of data collection, 1,477 days from commencing treatment. When controlling for immunophenotype, elevated PEA was significantly associated with shorter PFI (*p* = 0.042) with an increased hazards ratio (HR) of 1.816 [95% Confidence Interval (CI): 1.020 −3.232]. Elevated OEA was trending toward significance for shorter PFI (*p* = 0.090). There was no significant association between endocannabinoid level and PFI for AEA (*p* = 0.739) or total AG (*p* = 0.657).

The OST for all lymphoma patients was 252 days with an OST of 295 and 126 days for dogs with B cell lymphoma and T cell lymphoma, respectively. At the time of analysis, 33 dogs were dead and the remaining 7 were lost to follow up with a range of 182–1,148 days from initiation of chemotherapy. There were no significant associations between endocannabinoid level and OST (AEA: *p* = 0.664; OEA: *p* = 0.750; PEA: *p* = 0.846; total AG: *p* = 0.288).

## Discussion

The endocannabinoid system is a widely expressed entity that has many roles in the physiologic events that are required to maintain and restore cell and tissue homeostasis. This signaling system has several important roles in the pathways involved in tumor growth and progression which has been previously documented in DLBCL and CLL in humans (14, 15). Here we demonstrated for the first time significantly higher levels of OEA, AEA, and PEA in dogs with lymphoma compared to healthy controls. The involvement of the endocannabinoid system in tumor progression is still under investigation and this relationship can be challenging to elucidate as endocannabinoids can have both pro- and anti-cancer effects. However, endocannabinoids have been postulated to potentiate cancer cell proliferation and survival through a variety of mechanisms. Zhang et al. demonstrated that AEA has pro-angiogenic properties through potentiation of fibroblast growth factor 2, which stimulates endothelial cell proliferation (25). Additionally, it has been demonstrated that exogenous endocannabinoids applied at nanomolar concentrations can induce cancer cell proliferation in glioblastoma and lung carcinoma cell lines through activation of epidermal growth factor receptor (EGFR), a receptor tyrosine kinase commonly upregulated in cancer that can activate downstream pro-oncogenic signaling pathways resulting in cancer cell proliferation (26, 27).

Our data found that in opposition with the other measured endocannabinoids, total AG is higher in healthy control dogs compared to dogs with lymphoma. This contrasts with the study by Zhang et al. that found significantly elevated levels of 2-AG in humans with DLBCL (14). In aqueous environments 2-AG spontaneously isomerizes to 1-AG *via* acyl migration and can be challenging to measure independently (19, 23). Most methods assume that 2-AG and 1-AG both originate from biological 2-AG (19); therefore, in our study the peaks were measured together and summed to total AG. However, a recent study found that 1-AG is a biologically active compound with its own effects on CB1. Therefore, it is possible that measuring total AG instead of 2-AG led to the opposing results from Zhang et al. (14). Alternatively, this could imply that downregulation of the 2-AG signaling system is involved in the pathogenesis of canine multicentric lymphoma. Elevated levels of MAGL, which is the enzyme responsible for degradation of 2-AG, has been shown to be highly expressed in a variety of aggressive human cancer cells and primary tumors (28). Additionally, activation of CB1 by 2-AG has been demonstrated to induce cell apoptosis through activation of ERK 1/2 expression and increased ceramide synthesis (29). Therefore it is possible downregulation of 2-AG is contributing to the progression of canine multicentric lymphoma and further investigation is warranted to evaluate MAGL levels and CB1 activation.

We found a significant relationship between endocannabinoid levels and age, with decreasing endocannabinoid levels in older normal dogs and older dogs with T cell lymphoma. Interestingly, the reverse was true in dogs with B cell lymphoma, with increased endocannabinoids in older dogs with B cell lymphoma. It has been shown in both murine and human experimental and preclinical models that the endocannabinoid system varies with age. Although there has been some conflicting reports, studies have overall shown a reduction in CB1 stimulated activity and reduction in 2-AG with increasing age (30). It is thought that these changes are particularly linked to learning disturbances and sleep disorders (31, 32). The changes in endocannabinoid levels in patients with neoplasia have not been specifically evaluated in humans. In a study of patients with DLBCL, participants were age matched with their controls, thus the effect of age was not evaluated (14). Masoodi et al. found that there was no significant relationship between OEA levels and age in their control population or the CLL population; however, it is important to note the majority of their CLL patients were older with 97% being above 50 years and 80% above 60 years old (15). Our results contrast a study on canine chronic enteropathy by Febo et al. in which there was no significant association between endocannabinoids and age in healthy dogs (33). This may have been influenced by the age of the control group, the median age of our control group was 84 months as compared to 60 months in their population, however further research evaluating the impact of age is needed.

There were no correlations between endocannabinoid levels and lymphoma immunophenotype. In our population, the dogs with T cell lymphoma had a shorter PFI (79.5 days) compared to the dogs with B cell lymphoma (237 days) which is consistent with prior reports of shorter PFI in multicentric T cell lymphoma (1, 34). The lack of difference in the measured endocannabinoids suggests that they are not a factor responsible for the more aggressive biologic behavior of T cell lymphoma.

Elevated PEA levels were significantly associated with shortened PFI. PEA has not been previously evaluated in the context of human or canine lymphoma. PEA is an endogenous compound that has been primarily investigated for its anti-inflammatory properties, specifically its impact on reducing degranulation of mast cells in response to substance P (35). Mast cells have various roles in neoplasia and their accumulation can be either beneficial or detrimental based on the tumor microenvironment and the stage of disease. A protective role for mast cells has been demonstrated in early stages of human intestinal carcinoma where mast cells promote apoptosis (36). A similar finding was reported in canine mammary gland tumors where the density of stromal mast cell tumors predicted disease outcome, with higher metastatic rates in tumors with lower numbers of stromal mast cells (37). The literature with respect to lymphoma is conflicting but Hedstrom et al. found that mast cell infiltration is a favorable prognostic factor in people with DLBCL; patients with increased numbers of nodal mast cells had significantly better event-free survival (38). Studies in canine nodal lymphoma found significantly higher mast cell counts compared to control lymph nodes (39); however, they did not evaluate the prognostic implications. Overall, intratumoral mast cells may have a protective role in canine lymphoma that is being suppressed by elevated PEA. Alternatively, PEA has been shown to have both direct and indirect effects on a variety of different receptor targets (40) including CB2 and PPAR-α. Therefore, it is possible that PEA has other implications in canine lymphoma not related to mast cells and further investigation is needed.

OEA was found to be elevated in dogs with lymphoma compared to healthy controls. OEA is a naturally occurring ethanolamide lipid that acts as a satiety factor. OEA has been demonstrated to stimulate lipolysis in adipose tissue, bone marrow, and lymph nodes through its activation of PPAR-α (41) leading to increased levels of circulating free fatty acids. In humans, abnormalities in lipid metabolism have been previously reported in leukemia and lymphoma with increased levels of triglycerides and free fatty acids being demonstrated at diagnosis especially in patients with a high disease burden or bone marrow involvement (42, 43). Masoodi et al. found high levels of free fatty acids accompanied the elevated OEA levels in patients with CLL which correlated with OEA causing adipocyte lipolysis. They also found that adipocyte-derived lipid factors protected the CLL cells from chemotherapeutic drugs *in vitro* with increased cell survival when the CLL cells were cultured with adipocyte-conditioned media and vincristine as opposed to vincristine alone (15). Although it did not reach significance, OEA was trending toward being negatively associated with PFI. Therefore, it is possible that OEA may represent a non-invasive measurement of prognosis and a potential therapeutic target; however, further research is needed with increased number of dogs.

There was no significant association between the endocannabinoids measured and the OST in this population of dogs. Previous studies in humans have not evaluated the relationship between endocannabinoids and OST. OST can be challenging to evaluate in veterinary retrospective studies due to lack of standardization of the type and number of rescue protocols pursued. These differences may have influenced the OST in our population.

The limitations of this study are predominantly due to its retrospective nature. First, the majority of serum samples were obtained between 8:00 a.m. and 12:00 p.m.; however, some samples were obtained outside of this time frame. Although circadian rhythm had been shown to affect endocannabinoid levels in people and mice (44, 45), this has not been demonstrated specifically in dogs; however, in future studies this aspect should be considered and controlled. Additionally, the duration of storage of serum prior to the measurement of endocannabinoids was variable, raising the potential for inconsistency due to endocannabinoid breakdown. To date the stability in canine serum has not been validated, however a human study by Bobrich et al. found that AEA and 2-AG in serum were stable at 60 days when stored at −80°C (46). Thirdly, the control group was small and was not intentionally age or weight matched to the lymphoma population. For the lymphoma patients, full staging was not performed for all patients and substage was assigned retrospectively based on information in the medical record. The case management was not standardized, leading to some variation in treatment decision making and case follow up. Lastly, the endocannabinoid levels were only measured once at the time of diagnosis. Further studies with repeated endocannabinoid measurements throughout the CHOP protocol are necessary to evaluate the changes with respect to response to treatment and disease progression.

In conclusion, we found that OEA, AEA, and PEA levels are significantly higher in dogs with lymphoma compared to healthy controls at the time of diagnosis. Furthermore, elevated PEA at the time of CHOP chemotherapy initiation is associated with a shorter PFI. Further research into both the endocannabinoid receptors and the enzymes involved in their synthesis and degradation are needed to better elucidate the underlying mechanisms of this system and its effects on canine lymphoma. Additionally, prospective studies evaluating dynamic changes of endocannabinoids throughout the CHOP protocol and evaluation of potential therapeutic targets are warranted.

## Data Availability Statement

The raw data supporting the conclusions of this article will be made available by the authors, without undue reservation.

## Ethics Statement

The animal study was reviewed and approved by Animal Utilization Protocol #3603, University of Guelph, Guelph, ON, Canada. Written informed consent was obtained from the owners for the participation of their animals in this study.

## Author Contributions

SH: hypothesis generation, experimental design, organizing, and conducting the experiments. JH, SH, and GM: interpreting and analyzing the results. JH, SH, and JW: writing and revising the manuscript. All authors contributed to the article and approved the submitted version.

## Funding

Funding was provided by the Ontario Veterinary College Department of Clinical Sciences.

## Conflict of Interest

The authors declare that the research was conducted in the absence of any commercial or financial relationships that could be construed as a potential conflict of interest.

## Publisher's Note

All claims expressed in this article are solely those of the authors and do not necessarily represent those of their affiliated organizations, or those of the publisher, the editors and the reviewers. Any product that may be evaluated in this article, or claim that may be made by its manufacturer, is not guaranteed or endorsed by the publisher.
